# Predicted risk of heart failure pandemic due to persistent SARS-CoV-2 infection using a three-dimensional cardiac model

**DOI:** 10.1016/j.isci.2023.108641

**Published:** 2023-12-22

**Authors:** Kozue Murata, Akiko Makino, Keizo Tomonaga, Hidetoshi Masumoto

**Affiliations:** 1 Clinical Translational Research Program, RIKEN Center for Biosystems Dynamics Research, Kobe, Japan; 2 Laboratory of RNA Viruses, Department of Virus Research, Institute for Life and Medical Sciences, Kyoto University, Kyoto, Japan; 3 Department of Cardiovascular Surgery, Graduate School of Medicine, Kyoto University, Kyoto, Japan

**Keywords:** Health sciences, Medicine, Health technology, Natural sciences, Biological sciences, Cell biology, Stem cells research, Biology experimental methods

## Abstract

Patients with chronic cardiomyopathy may have persistent viral infections in their hearts, particularly with SARS-CoV-2, which targets the ACE2 receptor highly expressed in human hearts. This raises concerns about a potential global heart failure pandemic stemming from COVID-19, an SARS-CoV-2 pandemic in near future. Although faced with this healthcare caveat, there is limited research on persistent viral heart infections, and no models have been established. In this study, we created an SARS-CoV-2 persistent infection model using human iPS cell-derived cardiac microtissues (CMTs). Mild infections sustained viral presence without significant dysfunction for a month, indicating persistent infection. However, when exposed to hypoxic conditions mimicking ischemic heart diseases, cardiac function deteriorated alongside intracellular SARS-CoV-2 reactivation in cardiomyocytes and disrupted vascular network formation. This study demonstrates that SARS-CoV-2 persistently infects the heart opportunistically causing cardiac dysfunction triggered by detrimental stimuli such as ischemia, potentially predicting a post COVID-19 era heart failure pandemic.

## Highlights

• Persistent SARS-CoV-2 infection model of human cardiac tissue was established
• Hypoxic stress to the persistent infection model led to cardiac dysfunction
• ACE2 and SARS-CoV-2 S protein expression were elevated after the hypoxic stress
• This research may predict a “heart failure pandemic” in the post COVID-19 era

## Graphical abstract

**Figure:**
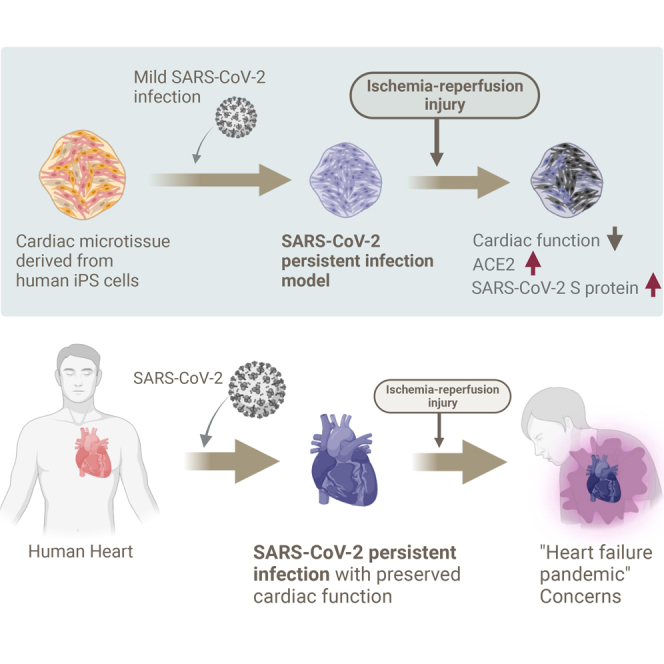

## Introduction

The cumulative number of people infected with the novel coronavirus (SARS-CoV-2) has exceeded 754 million and the number of deaths has reached 6.8 million as of February 2023. The dominant SARS-CoV-2 mutant strain continues to be the BA.5 strain (omicron strain) worldwide (WHO, Edition 130).^1^ Case fatality risk and hospitalization is reduced in the delta and omicron strains compared to earlier strains due to several factors such as a reduction in the inherent severity of the virus, an increase in spontaneous outbreaks, vaccination and the use of therapeutic agents (UKHSA, 2022).^2^ The trend has moved from “COVID-19 pandemic” to the “post COVID-19” which encourages us to focus not only on acute phase symptoms, but on chronic health problems.^3^

The expression of ACE2 in the heart is reported to be higher than in other organs.^4^ In addition, several literatures reported that ACE2 expression in cardiomyocyte is higher in patients with heart failure than in healthy controls.^5^^,^^6^^,^^7^ This suggests that the heart is susceptible to SARS-CoV-2 infection, which is more pronounced under detrimental conditions such as heart failure. More than 10 years before the COVID-19 pandemic, several researches revealed the detection of viral genomes in samples of endocardial biopsies from patients with idiopathic chronic cardiomyopathy suggesting that viral infection is deeply involved in the pathogenesis of the disease. These researches also suggest that chronic viral infection impairs cardiac function.^8^^,^^9^ Even though the persistent viral infection was recognized to be associated with the onset and the progress of chronic cardiomyopathy at that time, very limited research focusing on persistent viral heart infection had been reported before 2019, possibly because of the unawareness of its significance in healthcare. COVID-19 pandemic may have drastically changed the situation as the population at risk of future heart failure due to persistent infection of SARS-CoV-2 is expected to exponentially increase. Even though conclusive clinical evidence that persistent SARS-CoV-2 infection is associated with declined cardiac function has not been reported so far, the proof-of-concept study of the possibility of SARS-CoV-2 persistent infection of the heart and the potential risk of opportunistic progression of heart failure should be validated by a three-dimensional human cardiac tissue model which would serve as the alarm bell for a global healthcare risk.

## Result

### Establishment of SARS-COV-2 persistent infection model using human iPS cell-derived cardiac microtissues

**Figure 1:**
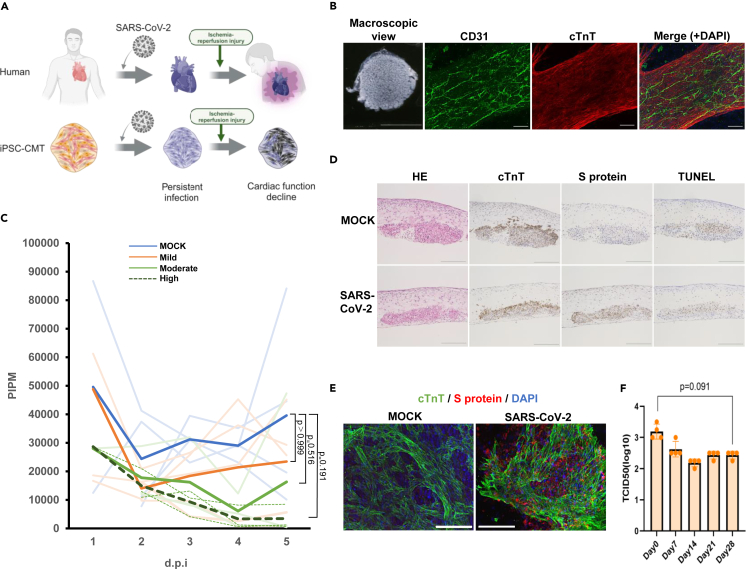
Persistent infection model of SARS-CoV-2 (A) Conceptual schema of the SARS-CoV-2 persistent infection model. (B) Vascular network-like structure of the CMT. (Left) Macroscopic observation of CMT. (Right) immunofluorescence analysis (IFA) of CMT for cTnT (green) and CD31 (red) after 7 days of rocking culture. (C) Assessment of cardiac function over time during persistent infection. PIPM: Pulsatile index per minute. Light lines indicate individual values; dark lines indicate average values. MOCK (no infection, blue: n = 4), Mild (orange: n = 5), Moderate (green: n = 3), High (dashed line: n = 3). (D) Histological analysis of CMT after 28 days of infection with SARS-CoV-2 in mild titers. HE: hematoxylin-eosin staining, cTnT: cardiac isoform of troponin-T, TUNEL: TdT-mediated dUTP nick-end labeling. (E) IFA of CMT for cTnT (green) and S protein (red) after 28 days of infection with SARS-CoV-2 in mild titers. (F) SARS-CoV-2 titers in culture medium supernatant from 7 to 28 d.p.i. n = 4, Error bars show S.D. Scale bars: 1cm in B (Left), 200 μm in B (Right), 100 μm in D and E. Nuclei were stained with 4′,6-diamidino-2-phenylindole (DAPI; blue).

In the present study, we experimentally demonstrated persistent heart infection with SARS-CoV-2 and cardiac dysfunction triggered by ischemic injury using human iPS cell-derived cardiac microtissues (CMTs) (Figure 1A, Video S1). The CMTs are composed of cardiomyocytes and other cardiac component cells (vascular endothelial cells and vascular mural cells) differentiated from human iPS cells^10^ and yield a vascular network-like structure that morphologically and functionally mimics the human heart^11^ (Figure 1B). The CMTs were infected with SARS-CoV-2 (SARS-CoV-2/UT-NCGM02/Human/2020/Tokyo), and the cardiac function was evaluated by a video-based method to evaluate tissue contractility.^12^ At 7 days post-infection (d.p.i), mild, moderate, and high titers showed functional deterioration compared to that in the non-infected group (MOCK) (Figure 1C); indicating that viral infection can lead to the reduction of tissue contractility in the acute phase of viral infection. Intriguingly, mild and moderate titers showed a recovery trend in cardiac function at 28 d.p.i., whereas high titers showed a sustainable decrease of contractility without recovery which may indicate a clinical case of the deterioration of cardiac function during the acute phase of COVID-19 requiring heart transplantation.^13^ Histological analysis and Immunofluorescent analysis (IFA) for CMTs infected with mild titers revealed the expression of SARS-CoV-2-derived spike protein (S protein; recognized by the host receptor) which was co-localizing with cardiomyocytes at 28 d.p.i. (Figures 1D and 1E). Nevertheless, the apoptosis of cardiomyocytes was the same level as that in MOCK and the structure of cardiac isoform of troponin T (cTnT), the troponin complex of cardiomyocytes, was maintained (Figure 1D). Measurement of viral titer using VeroE6 cells showed high viral titers up to 28 d.p.i., suggesting that persistently infected viruses retained their amplification ability and maintained infectivity to adjacent tissues (Figure 1F). IFA revealed that this persistent infection is partly present in endothelial cells as well as in cardiomyocytes, while vascular network formation was slightly disrupted only where strong co-localization with S protein and CD31, a maker of cardiac endothelial cells,^14^ was detected (Figure S1). These results indicate that mild infection can cause persistent SARS-CoV-2 infection of cardiac tissue without causing functional impairment.

Video S1. Microscopic observation of pulsating cardiac microtissues, related to Figure 1

### Hypoxic stress under persistent SARS-COV-2 infection causes cardiac dysfunction

**Figure 2:**
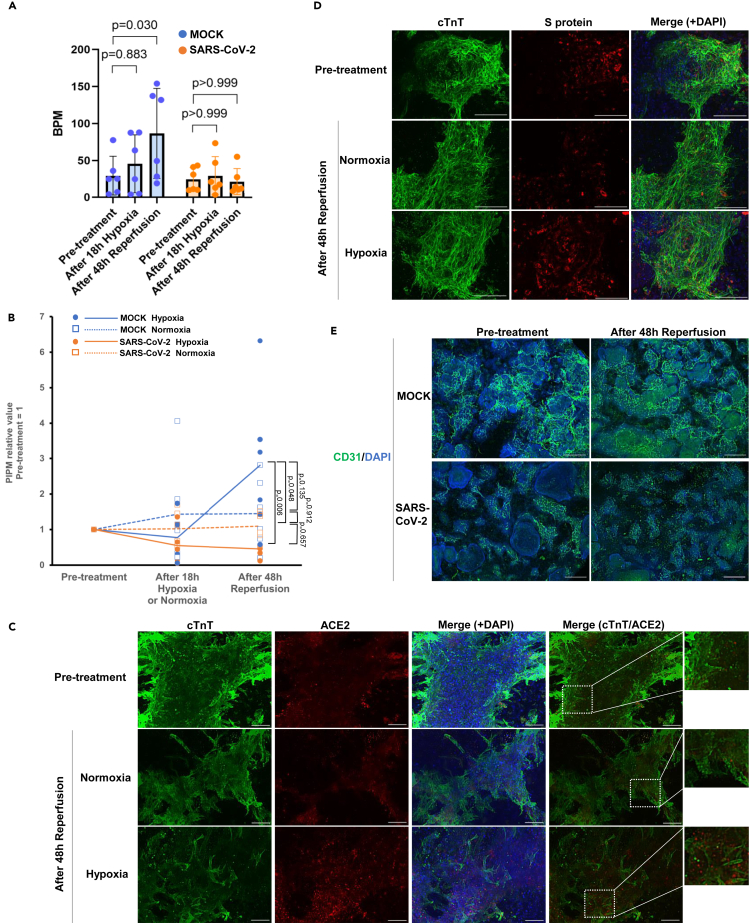
Deterioration of the tissue function of persistent infection model of SARS-CoV-2 triggered by hypoxic stress (A and B) Assessment of cardiac function. “SARS-CoV-2” indicates persistent infection model of SARS-CoV-2. (A) Beats per minute (BPM) at pre-treatment and after 48h of reperfusion condition (n = 6 each). Error bars show S.D. B, PIPM over time (n = 6 each). (C and D) IFA for persistent infection model of SARS-CoV-2 before treatment (Pre-treatment), 18h of hypoxia treatment (Hypoxia) or Normoxia followed by 48h reperfusion treatment. (C) cTnT (Green) and ACE2 (Red). (D) cTnT (Green) and S protein (Red). (E) CD31 (Green). Scale bars: C,D,100 μm. (E) 500 μm. Nuclei were stained with DAPI (Blue).

The persistent infection model experimentally indicated that a group of patients with history of mild SARS-CoV-2 infection who developed slight cardiac dysfunction may exist, and that global cardiac function is compensated for and may superficially be maintained without developing heart failure. However, such patients would be at marginal risk of heart failure and could opportunistically develop cardiac dysfunction and eventually heart failure under additional cardiac stress (Figure 1A). To experimentally validate this hypothesis, both the CMT model with (SARS-CoV-2) and without (MOCK) persistent infection were exposed to hypoxic stress mimicking ischemic heart disease. The hypoxic condition used in this study was a low-oxygen environment (<0.1% oxygen concentration) created by a hypoxic cultivation system. The treatment with hypoxic stress for 18 h and subsequent normoxic condition for 48 h resulted in increased pulsating frequency and contractile functional recovery in the MOCK. However, the persistently infected CMTs with SARS-CoV-2 exhibited no increase in pulsating frequency and showed further deterioration of the contractile function (Figures 2A and 2B Videos S2 and S3). IFA revealed that intracellular ACE2 expression in cardiomyocytes was upregulated by the hypoxia and reperfusion (Figure 2C). Accordingly, co-localization of cTnT and S protein was facilitated by the hypoxia, indicating intracellular reactivation of SARS-CoV-2 (Figure 2D). Nevertheless, the viral titer was not increased by the hypoxia which may imply the manner of the reactivation of SARS-CoV-2 which would not increase infectivity to other tissue but boost intracellular activity of SARS-CoV-2 (Figure S2). IFA for CD31 revealed that vascular network formation was globally fragmented in persistent infection model with SARS-CoV-2 after ischemia and reperfusion, whereas it was maintained in MOCK (Figure 2E). These results indicate that the hypoxic stress disrupted vascular network formation possibly because of the reactivation of SARS-CoV-2 and conferred functional deterioration of the infected CMTs.

Video S2. Microscopic observation of pulsating cardiac microtissues without infection of SARS-CoV-2 before and after hypoxia-reperfusion, related to Figure 2

Video S3. Microscopic observation of pulsating cardiac microtissues with persistent infection of SARS-CoV-2 before and after hypoxia-reperfusion, related to Figure 2

### The hypoxic stress under persistent SARS-CoV-2 infection does not cause the upregulation of inflammatory cytokines

**Figure 3:**
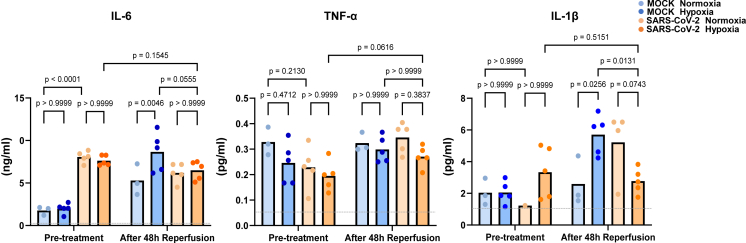
Measurement of cytokine levels in the culture supernatant before and after hypoxia-reperfusion treatment in cardiac microtissues by ELISA **Left:** Measurement of IL-6 in supernatant. **Middle:** Measurement of TNF-α in supernatant. **Right:** Measurement of IL-1β in supernatant. N = 3–5. Gray dashed lines represent the detection limits (IL-6: 0.7 pg/mL, TNF-α: 0.049 pg/mL, IL-1β: 1 pg/mL). The values under the detection limits are not shown.

To investigate whether the upregulation of inflammatory cytokines plays a role in the decreased cardiac function when additional hypoxic stress is applied to the SARS-CoV-2 persistent infection model, we quantified cytokine levels in the culture supernatant before and after subjecting them to hypoxic stress using enzyme-linked immunosorbent assay (ELISA). We specifically focused on interleukin (IL)-6, tumor necrosis factor (TNF)-α, IL-1β, and interferon (IFN)-γ as representative cytokines associated with cytokine storms in COVID-19.^15^^,^^16^ Our findings revealed that the expression levels of IL-6, TNF-α and IL-1β did not increase under hypoxic stress in the context of persistent infection (Figure 3). Additionally, most samples exhibited values near or below the detection limit for IFN-γ, regardless of the presence of persistent SARS-CoV-2 infection, and showed no increase under hypoxic stress in persistently infected tissues (Figure S3). These results suggest that the reduced cardiac function observed under hypoxic stress in the context of persistent infection, as shown in Figures 2A and 2B, is not dependent on the expression level of cytokines.

## Discussion

The human iPS cell-based cardiac tissue model established in the present study is the first report to experimentally demonstrate SARS-CoV-2 persistent infection of the human heart exhibiting functional deterioration caused by the opportunistic intracellular reactivation of viral infection. We experimentally demonstrated that cardiac tissues under persistent infections with SARS-CoV-2 are at high risk of cardiac dysfunction with additional hypoxic stress (Figure 2B). In other words, the explosive increase in the number of virus-infected patients due to the COVID-19 pandemic may have led to an enormous increase in the number of patients at potential risk for future heart failure. These patients would be predicted to maintain cardiac function superficially despite being at marginal risk. In clinical practices, such high-risk patients should be identified by detecting the virus itself or the viral genome in endocardial biopsy tissue or by monitoring blood troponin levels. According to our study, cardiac dysfunction associated with persistent infection was the result of increased ACE2 expression in cardiomyocytes in response to additional stress (Figure 2D), reactivation of SARS-CoV-2 in cardiomyocyte (Figure 2C), and disruption of the vascular network-like structure (Figure 2E). Hence, apart from viral clearance from the heart, strategies that can inhibit these processes are being considered as potential therapeutic approaches.

In COVID-19, a common factor contributing to severe outcomes is the occurrence of a 'cytokine storm', characterized by an abnormal increase in inflammatory cytokines like IL-6, TNF-α, IL-1β, and IFN-γ.^15^^,^^16^ However, the established persistent SARS-CoV-2 infection model in this study, which lacks immune-related cells, could not fully replicate the impact of cytokine storms typically mediated through immune cells. This limitation emphasizes the need for further research to validate these aspects once a persistent infection model involving immune factors is developed in the future. On the other hand, as shown in Figure 3, we observed that hypoxic stress did not lead to the upregulation of representative inflammatory cytokines associated with cytokine storms in our persistently infected model, despite hypoxic stress causing cardiac functional deterioration (Figures 2A and 2B). These results suggest that the functional decline was not driven by cytokines involved in inflammatory responses, and it may indicate the potential for SARS-CoV-2 infection to directly affect cardiac function. Intriguingly, we observed that the level of IL-6 was higher in cases of persistent SARS-CoV-2 infection compared to the non-infected control group prior to the initiation of hypoxic treatment (Figure 3), whereas other measured cytokines did not show an upregulation due to the persistent infection. In animal models, such as mice and hamsters, SARS-CoV-2 infection or the introduction of viral proteins led to increased expression of INF-γ, IL-6, TNF-α, and IL-1β.^17^^,^^18^ Furthermore, large cohort studies have revealed elevated levels of IL-1β, IL-6, and TNF in the serum of clinical patients with an average period of 8 months after infection.^19^ Our findings may suggest that the persistent SARS-CoV-2 infection model established in this study partially replicates certain aspects of the immune response *in vivo*, even though it is not complete, possibly due to the lack of inflammatory immune cells. In the future, it is conceivable that addressing this incompleteness in replicating the pathological condition could be achieved through co-culturing with immune cells, leading to a more comprehensive understanding of cardiac dysfunction induced by SARS-CoV-2.

Furthermore, this system poses challenges when considering the route of viral infection in heart tissue, as it requires considering the complex interaction between multiple organs. Currently, organoid research is progressing rapidly aiming for the recapitulation of the structure and function of various organs, including the heart, nerves, lungs, and liver.^20^ The development of a multi-organ model, such as MultiCUBE platform,^21^ which reproduces the biological processes that occur in the human body as organ-on-a-chip would greatly aid in the investigation of interactions between different organs and potentially help unravel the possible route of SARS-CoV-2 infection to the heart in the future.

In conclusion, this report may serve as a warning for the possibility of a heart failure pandemic in the post COVID-19 era. As a countermeasure against this global healthcare risk, this model would serve as a useful tool to investigate the mechanism of the onset and the progression of SARS-CoV-2 cardiomyopathy and to develop therapeutic options.

### Limitations of the study

While this study provides valuable insights into SARS-CoV-2 persistent infection in cardiac tissues, it is crucial to acknowledge specific limitations that may affect the interpretation of our findings. The findings are derived from *in vitro* model created from human iPS cells, offering significant insights but potentially not fully representing the diverse factors within the human body. Phenomena observed under hypoxic stress may be influenced by intricate interactions among different tissues and organs, necessitating further investigation. Furthermore, the necessity to conduct the entire series of experiments in a P3 level safety laboratory resulted in a relatively small sample size, potentially limiting the generalizability of our results. These limitations underscore the importance of future research endeavors to validate and further extend our findings.

## STAR★Methods

### Key resources table

**Table:** 

REAGENT or RESOURCE	SOURCE	IDENTIFIER
**Antibodies**		

anti-CD144, R-PE, Clone: 55-7H1,	BD Biosciences	Catalog # 560410; RRID:AB_1645502
anti-CD140b, R-PE, Clone: 28D4	BD Biosciences	Catalog # 558821; RRID:AB_397132
anti-Troponin T, Cardiac Isoform Ab-1, Clone: 13-11	Thermo Fisher Scientific	Catalog # MS-295-P0; RRID:AB_61807
SARS-CoV-2 spike glycoprotein (S protein) monoclonal mouse antibody	Osaka University	N/A
Human CD31/PECAM-1 Antibody; CD31 (monoclonal mouse IgG1, clone 9G11)	R&D	Catalog # BBA7; RRID:AB_356960
Anti-ACE2 Polyclonal Antibody	Bioss	Catalog # bs-1004R; RRID:AB_10856553
Anti-SARS-CoV-2 spike glycoprotein antibody	Abcam	Catalog # ab272504; RRID:AB_2847845
Goat anti-Rabbit IgG (H+L) Cross-Adsorbed Secondary Antibody, Alexa Fluor 488	Thermo Fisher Scientific	Catalog # A-11008; RRID:AB_143165
Goat anti-Mouse IgG (H+L) Cross-Adsorbed Secondary Antibody, Alexa Fluor 546	Thermo Fisher Scientific	Catalog # A- 11003; RRID:AB_2534071
Goat anti-Mouse IgG (H+L) Cross-Adsorbed Secondary Antibody, Alexa Fluo 488	Thermo Fisher Scientific	Catalog # A-11001; RRID:AB_2534069

**Bacterial and virus strains**		

SARS-CoV-2/UT-NCGM02/Human/2020/Tokyo (EPI_ISL_418809)	The University of Tokyo	N/A

**Chemicals, peptides, and recombinant proteins**		

StemFit AK02N medium	AJINOMOTO	Catalog #AK02N
iMatrix-511 silk	FUJIFILM Wako	Catalog # 387-10131
CultureSure (R) Y-27632 (25mg)	FUJIFILM Wako	Catalog #034-24024
Recombinant human bFGF 100ug	FUJIFILM Wako	Catalog # 060-04543
Human recombinant VEGF 10ug	FUJIFILM Wako	Catalog # 223-01311
Growth factor reduced Matrigel	Corning	Catalog # 356231
Fetal Bovine Serum (FBS)	Corning	Catalog # 35-079-CV
RPMI 1640	Thermo Fisher Scientific	Catalog # 21870092
TrypLE Select	Thermo Fisher Scientific	Catalog # A1285901
B27 supplement minus insulin 10ml	Thermo Fisher Scientific	Catalog # A1895601
Alpha minimum essential medium	Thermo Fisher Scientific	Catalog # 11900024
DAPI (4‘,6-diamidino-2-phenylindole)	Thermo Fisher Scientific	Catalog # D1306
Dulbecco's modified Eagle’s medium (DMEM)	Thermo Fisher Scientific	Catalog # 11885084
Fetal calf serum (FCS)	Thermo Fisher Scientific	Catalog # A5256701
Recombinant Human/Mouse/Rat ActivinA (50μg)	R&D	Catalog # 338-AC-050
BMP4, recombinant (10μg)	R&D	Catalog # 314-BP-010
CHIR99021 10mg	TOCRIS	Catalog # 4423/10
IWP-4 2mg	REPROCELL	Catalog # 04-0036-base
XAV939 10mg	Merck	Catalog # 575545-10MGCN
Accutase	Nacalai Tesque	Catalog # 1267954
Saponin	Nacalai Tesque	Catalog # 30502-42
Penicillin-Streptomycin Mixed Solution	Nacalai Tesque	Catalog # 26253-84

**Critical commercial assays**		

BIO-NIX system	SUGIYAMA-GEN CO.	Catalog # nBIONIX-3
LIVE/DEAD fixable Aqua dead cell staining kit	Thermo Fisher Scientific	Catalog # L34957
Zenon® APC Mouse IgG1 Labeling Kit	Thermo Fisher Scientific	Catalog # Z25051
*in situ* Apoptosis Detection Kit	Takara Bio Inc	Catalog # MK500
Avidin-biotin-peroxidase complex (ABC)	Vector Labotarories	Catalog # PK-6103
Human IL-6 Quantikine ELISA Kit	R&D	Catalog # D6050
Human TNF-alpha Quantikine HS ELISA	R&D	Catalog #HSTA00E
Human IL-1 beta/IL-1F2 Quantikine ELISA Kit	R&D	Catalog # DLB50
Human IFN-gamma Quantikine ELISA Kit	R&D	Catalog # DIF50C

**Experimental models: Cell lines**		

Human iPSCs lines 201B6	the Center for iPS Cell Research and Application (CiRA), Kyoto University, Kyoto, Japan	N/A
VeroE6/TMPRSS2	National Institutes of Biomedical Innovation, Health and Nutrition, Osaka, Japan	Catalog # JCRB1819

**Software and algorithms**		

CytExpert software	Beckman Coulter,	N/A
BZ-X800E sectioning functions	Keyence	N/A
MUSCLEMOTION	Open source	N/A
Image J	Open source	N/A
Prism 9	GraphPad	N/A

**Other**		

UpCell plate	CellSeed	Catalog # CS3003
Compact Digital Rocker	DLAB	Catalog # 3-7044-09
iP-TEC® live cell transportation system	SANPLATEC CO	Catalog # 28460
CytoFLEX S	Beckman Coulter,	N/A

### Resource availability

#### Lead contact

Further information and requests for resources and reagents should be directed to and will be fulfilled by the lead contact, Hidetoshi Masumoto (hidetoshi.masumoto@riken.jp).

#### Materials availability

This study did not generate new unique reagents.

#### Data and code availability

• Date: Data reported in this paper will be shared by the lead contact upon request.
• Code: This paper does not report original code.
• All other requests: Any additional information required to reanalyze the data reported will be shared by the lead contact upon request.

### Experimental model and study participant details

#### Cell lines

Human iPSCs lines 201B6 was established at the Center for iPS Cell Research and Application (CiRA), Kyoto University, Kyoto, Japan from skin fibroblasts of a female human.^22^ Detailed culture methods are described in Method details. The VeroE6/TMPRSS2 cell line (JCRB1819) was obtained from the JCRB cell bank of the National Institute of Biomedical Innovation, Health and Nutrition, Osaka, Japan, and was cultured in Dulbecco's modified Eagle’s medium (DMEM) (Thermo Fisher Scientific) containing 10% fetal calf serum (FCS) (Thermo Fisher Scientific) and 1% penicillin-streptomycin (Nacalai tesque, Kyoto, Japan) in 5% CO_2_ atmosphere at 37°C.

### Method details

#### Data reporting

No statistical methods were used to predetermine sample size because of rather limited number of experiments with P3 biosafety level using SARS-CoV-2. All results were confirmed with >2 repetitive independent experiments.

#### Maintenance of human iPS cells (iPSCs) and differentiation of cardiovascular cell lines

The present study used Human iPSCs lines 201B6 established at the Center for iPS Cell Research and Application (CiRA), Kyoto University, Kyoto, Japan.^22^ The maintenance of human iPSCs and differentiation of cardiovascular cells was conducted in accordance with our previous studies^10^^,^^11^^,^^23^ with modifications. In brief, iPSCs were expanded and maintained with StemFit AK02N medium (AJINOMOTO, Tokyo, Japan). At confluence, the cells were dissociated with TrypLE Select (Thermo Fisher Scientific, Waltham, MA, USA), dissolved in 0.5 mM ethylenediaminetetraacetic acid in PBS (1:1) and passaged as single cells (5,000 – 8,000 cells/cm^2^) every 7 days in AK02N containing iMatrix-511 silk (FUJIFILM Wako Pure Chemical Corp., Osaka, Japan) (0.125 μg/cm^2^) (uncoated laminin fragment^24^) and ROCK inhibitor (Y-27632, 10 μM, FUJIFILM Wako). For cardiovascular cell differentiation, single iPSCs were seeded onto Matrigel-coated plates (1:60 dilution) at a density of 300,000–400,000 cells/cm^2^ in AK02N with Y-27632 (10 μM). At confluence, the cells were covered with Matrigel (Corning, Corning, NY, USA) (1:60 dilution in AK02N) one day before induction. We replaced the AK02N medium with RPMI + B27 medium (RPMI 1640, Thermo Fisher; 2 mM L-glutamine, Thermo Fisher; 1× B27 supplement without insulin, Thermo Fisher) supplemented with 100 ng/mL Activin A (R&D, Minneapolis, MN, USA) (differentiation day 0; d0) and 5 μM CHIR99021 (Tocris Bioscience, Bristol, UK) was added for 24h, which was followed with supplementation with 10 ng/mL bone morphogenetic protein 4 (BMP4; R&D) and 10 ng/mL basic fibroblast growth factor (bFGF; FUJIFILM Wako) (d1) for 4 days without culture medium change. At d5, the culture medium was replaced with RPMI1640 medium supplemented with 50 ng/ml of vascular endothelial cell growth factor (VEGF)165 (FUJIFILM Wako), 2.5 μM IWP4 (REPROCELL, Beltsville, MD, USA) and 5 μM XAV939 (Merck, Kenilworth, NJ, USA). The culture medium was refreshed with RPMI1640 supplemented with 50 ng/ml VEGF every other day. Beating cells appeared at d11 to d15. In this protocol, we could exclusively induce cardiomyocytes (CMs) and vascular endothelial cells (ECs). To control the percentages of vascular mural cells (MCs) sufficient to form cell sheets, we used a part of differentiation culture for MC differentiation and induced the differentiation of MCs as required; after d3, the culture medium was replaced with RPMI + FBS medium [RPMI1640, 2 mM of L-glutamine, 10% fetal bovine serum (FBS)] and was refreshed every other day.

#### Generation of cardiac microtissues (CMT)

After differentiation (d13), cells were dissociated by incubation with Accutase (Nacalai Tesque, Kyoto, Japan), and the cell population was measured by flow cytometry of the mixture of cells. The cell mixture was plated onto an FBS-coated 12-well Multi-well UpCell plate (CellSeed, Tokyo, Japan) at 3,600,000 cells/well with 2 mL of attachment medium [AM; alpha minimum essential medium (αMEM) (Thermo Fisher) supplemented with 10% FBS and 5 × 10^−5^ M of 2-mercaptoethanol] containing 25 ng/ml VEGF and 10 μM of Y-27632. After two days in culture, 25 ng/ml VEGF was added to the culture medium. After two more days in culture, the cells were moved to room temperature. Within 15 min, the cells detached and floated in the media as monolayer cardiac tissue sheets.^10^ The collected cardiac tissue sheets were allowed to reattach to Matrigel-coated dishes and incubated with AM containing 50 ng/ml Y-27632 for 24h to culture on a Compact Digital Rocker (DLAB, Beijing, China) at 60 rpm and 13 degrees for 14-28 days.^11^

#### Virus preparation

The CMTs were prepared at RIKEN, Kobe, Japan and transferred to Kyoto University, Kyoto, Japan using iP-TEC® live cell transportation system (SANPLATEC CO., Ldd., Osaka, Japan) within 2h. SARS-CoV-2 strain used in this study, SARS-CoV-2/UT-NCGM02/Human/2020/Tokyo (EPI_ISL_418809), was isolated from a mild case at the University of Tokyo.^25^ The virus was propagated in VeroE6/TMPRSS2 cells and purified through ultracentrifugation with 20% sucrose at 27,000 rpm for 2h at 4°C. The pellets were suspended in Tris-EDTA buffer (pH 8.0) and centrifuged at 3,000 rpm for 10 min at 4°C. SARS-CoV-2 titer was determined by 50% tissue culture infectious dose (TCID50) of VeroE6/TMPRSS2. All experiments using SARS-CoV-2 were performed in biosafety level 3 containment laboratory at Kyoto University, Japan which was registered to use to the Ministry of Agriculture Forestry and Fisheries, Japan.

#### Development of persistent infection model

For the establishment of persistent infection with SARS-CoV-2, we used a dynamic rocking culture as previously reported.^3^ We infected the CMTs with SARS-CoV-2 at titers MOI-30 (Mild), MOI-100 (Moderate), and MOI-300 (High) measured in VeroE6/TMPRSS2 cells and cultured on a Compact Digital Rocker (Thermo Fisher) at 60 rpm and 13 degrees for 14-28 days. The medium was refreshed with AM once every 4 days. The non-infected group was described as MOCK in which AM without viruses was used for the treatment.

#### Hypoxic stress treatment

The CMT model, including one group with persistent infection of SARS-CoV-2 (referred to as SARS-CoV-2) and another group without infection (referred to as MOCK), was subjected to a hypoxic condition with <0.1% oxygen concentration monitoring with an O_2_ meter included in the BIO-NIX system (nBIONIX-3, SUGIYAMA-GEN CO., Tokyo, Japan) for 18 hours, which will be referred to as Hypoxia. The control group was maintained under normal oxygen concentration, described as Normoxia. Following the hypoxic/normoxic treatment, both groups were returned to a normal oxygen concentration and incubated for 48 hours under rocking culture.

#### Flow cytometry

Flow cytometry was conducted in accordance with our previous study with modifications.^1^ Differentiated cardiovascular cells and cardiac tissue sheets were dissociated by incubation with Accutase and stained with one or a combination of the following surface markers: anti-PDGFRβ conjugated with phycoerythrin (PE), clone 28d4, 1:100 (BD, Franklin Lakes, NJ, USA) for MCs, and anti-VE-cadherin conjugated with phycoerythrin (PE), clone 55-7h1, 1:100 (BD) for ECs. To eliminate dead cells, cells were stained with the LIVE/DEAD fixable Aqua dead cell staining kit (Thermo Fisher). For cell surface markers, staining was carried out in PBS with 5% FBS. For intracellular proteins, staining was carried out in cells fixed with 4% paraformaldehyde (PFA) in PBS. Cells were stained with the anti-cardiac isoform of troponin T (cTnT) (clone 13-11) (Thermo Fisher) labelled with APC using Zenon technology (Thermo Fisher) (1:50) for CMs. The staining was performed in PBS with 5% FBS and 0.75% saponin (Nacalai Tesque). The stained cells were analyzed by CytoFLEX S (Beckman Coulter, Brea, CA, USA). Data was collected from at least 10,000 events. Data was analyzed with CytExpert software (Beckman Coulter). The percentage of CMs, ECs and MCs in cardiac tissue sheets used in the present study was as follows: CM, 35.53±10.47 %, EC, 1.86±0.93 %, MC, 37.43±7.07 % (n=6).

#### Histological analysis and immunohistochemical staining

CMTs were fixed in 4% PFA and embedded in paraffin. Sections (6-μm thickness) were prepared and stained with hematoxylin-eosin and TdT-mediated dUTP Nick-End Labeling (TUNEL; *in situ* Apoptosis Detection Kit) (Takara Bio Inc., Kusatsu, Japan; according to product protocols). For immunohistochemical staining, paraffin sections were stained first with antibodies for cTnT (Thermo Fisher) (1:100) and SARS-CoV-2 spike glycoprotein (S protein) [monoclonal mouse antibody provided by Dr. Hisashi Arase (Osaka University)] (1:50). They were incubated with biotinylated second antibody (1:300) and Avidin-biotin-peroxidase complex (ABC) (ABC-Elite, Vector Labotarories, 1:100). Coloring reaction was carried out with DAB and nuclei were counterstained with hematoxylin. All results were confirmed with >2 repetitive independent experiments.

#### Immunofluorescence analysis (IFA)

For IFA, CMTs were stained with cTnT antibody (Thermo Fisher) (1:250), CD31 (monoclonal mouse IgG1, clone 9G11) (R&D) (1:250), ACE2 (polyclonal antibody) (Bioss, Woburn, MA, USA) (1:250), S protein (ab272504), Abcam (1:250) or monoclonal mouse antibody provided by Dr. Hisashi Arase (Osaka University) (1:50), with DAPI (4‘,6-diamidino-2-phenylindole) (Thermo Fisher) (1:1000). Anti-mouse Alexa 546 (Thermo Fisher), anti-rabbit Alexa 488 (Thermo Fisher) and anti-mouse Alexa Fluor 488 (Thermo Fisher) were used as secondary antibodies. The tissues were photographed with an all-in-one fluorescence microscopic system, BZ-X800E (Keyence, Osaka, Japan) Combined Z-stack and sectioning functions. All results were confirmed with >2 repetitive independent experiments.

#### MUSCLEMOTION analysis (video-based method to evaluate tissue contractility)

MUSCLEMOTION is a versatile open-source software with a video-based system used to evaluate contractile function.^12^ We used the software as the provider instructed. In brief, we used ImageJ software and installed MUSCLEMOTION as a plug-in. The motion amplitude was used for analysis. Pulsatile index per minute (PIPM) represents the change in pulsatile force per minute; calculated using beats per minute (BPM) and pixel change.

#### Enzyme-linked immunosorbent assay (ELISA)

Before ELISA, thawed culture medium were centrifuged at 3,000 rpm for 5 min at 4°C to remove cell debris. The levels of IL-6, TNF-α, IL-1β and IFN-γ in the culture medium supernatant were assessed using Human IL-6 Quantikine ELISA Kit, Human TNF-alpha Quantikine HS ELISA, Human IL-1 beta/IL-1F2 Quantikine ELISA Kit, and Human IFN-gamma Quantikine ELISA Kit (R&D) following the manufacturer’s instructions. Absorbance at 450 nm was measured using the iMark microplate Absorbance Spectrophotometer (Bio-Rad, Philadelphia, PA, USA).

### Quantification and statistical analysis

All statistical analysis was performed using Prism 9 (GraphPad, Boston, MA, USA) by one-way ANOVA or Kruskal-Wallis test with two-tailed P values, assuming parametric data for samples ≥ 6 and nonparametric data for samples < 6. The multiple comparisons were conducted using the Bonferroni method. No blinded tests were used to procedure the samples.

## Supplemental information

Document S1. Figures S1‒S3

## References

[1] World Health Organization (2022).

[2] UK Health Security Agency (2022).

[3] Centers for Disease Control and Prevention (2022).

[4] Hikmet F., Méar L., Edvinsson Å., Micke P., Uhlén M., Lindskog C. (2020). The protein expression profile of ACE2 in human tissues. Mol. Syst. Biol..

[5] Tucker N.R., Chaffin M., Bedi K.C., Papangeli I., Akkad A.D., Arduini A., Hayat S., Eraslan G., Muus C., Bhattacharyya R.P. (2020). Myocyte-specific upregulation of ACE2 in cardiovascular disease: Implications for SARS-CoV-2-mediated myocarditis. Circulation.

[6] Chen L., Li X., Chen M., Feng Y., Xiong C. (2020). The ACE2 expression in human heart indicates new potential mechanism of heart injury among patients infected with SARS-CoV-2. Cardiovasc. Res..

[7] Guo J., Wei X., Li Q., Li L., Yang Z., Shi Y., Qin Y., Zhang X., Wang X., Zhi X., Meng D. (2020). Single-cell RNA analysis on ACE2 expression provides insights into SARS-CoV-2 potential entry into the bloodstream and heart injury. J. Cell. Physiol..

[8] Kühl U., Pauschinger M., Noutsias M., Seeberg B., Bock T., Lassner D., Poller W., Kandolf R., Schultheiss H.P. (2005). High prevalence of viral genomes and multiple viral infections in the myocardium of adults with “idiopathic” left ventricular dysfunction. Circulation.

[9] Kühl U., Pauschinger M., Seeberg B., Lassner D., Noutsias M., Poller W., Schultheiss H.P. (2005). Viral persistence in the myocardium is associated with progressive cardiac dysfunction. Circulation.

[10] Masumoto H., Ikuno T., Takeda M., Fukushima H., Marui A., Katayama S., Shimizu T., Ikeda T., Okano T., Sakata R., Yamashita J.K. (2014). Human iPS cell-engineered cardiac tissue sheets with cardiomyocytes and vascular cells for cardiac regeneration. Sci. Rep..

[11] Abulaiti M., Yalikun Y., Murata K., Sato A., Sami M.M., Sasaki Y., Fujiwara Y., Minatoya K., Shiba Y., Tanaka Y., Masumoto H. (2020). Establishment of a heart-on-a-chip microdevice based on human iPS cells for the evaluation of human heart tissue function. Sci. Rep..

[12] Sala L., Van Meer B.J., Tertoolen L.G.J., Bakkers J., Bellin M., Davis R.P., Denning C., Dieben M.A.E., Eschenhagen T., Giacomelli E. (2018). Musclemotion: A versatile open software tool to quantify cardiomyocyte and cardiac muscle contraction *in vitro* and *in vivo*. Circ. Res..

[13] Gill G., Roach A., Rowe G., Emerson D., Kobashigawa J., Lobo E.P., Esmailian F., Bowdish M.E., Chikwe J. (2023). Heart transplantation for COVID-19 myopathy in the United States. J. Heart Lung Transplant..

[14] Raniga K., Vo N.T.N., Denning C., Benest A.V. (2022). Methods Mol Biol.

[15] Mills R.J., Humphrey S.J., Fortuna P.R.J., Lor M., Foster S.R., Quaife-Ryan G.A., Johnston R.L., Dumenil T., Bishop C., Rudraraju R. (2021). BET inhibition blocks inflammation-induced cardiac dysfunction and SARS-CoV-2 infection. Cell.

[16] Hirano T., Murakami M. (2020). COVID-19: A New Virus, but a Familiar Receptor and Cytokine Release Syndrome. Immunity.

[17] Pan P., Shen M., Yu Z., Ge W., Chen K., Tian M., Xiao F., Wang Z., Wang J., Jia Y. (2021). SARS-CoV-2 N protein promotes NLRP3 inflammasome activation to induce hyperinflammation. Nat. Commun..

[18] Nouveau L., Buatois V., Cons L., Chatel L., Pontini G., Pleche N., Ferlin W.G. (2021). Immunological analysis of the murine anti-CD3-induced cytokine release syndrome model and therapeutic efficacy of anti-cytokine antibodies. Eur. J. Immunol..

[19] Schultheiß C., Willscher E., Paschold L., Gottschick C., Klee B., Henkes S.S., Bosurgi L., Dutzmann J., Sedding D., Frese T. (2022). The IL-1β, IL-6, and TNF cytokine triad is associated with post-acute sequelae of COVID-19. Cell Rep. Med..

[20] Yang S., Hu H., Kung H., Zou R., Dai Y., Hu Y., Wang T., Lv T., Yu J., Li F. (2023). Organoids: The current status and biomedical applications. MedComm.

[21] Suthiwanich K., Hagiwara M. (2023). Localization of Multiple Hydrogels with MultiCUBE Platform Spatially Guides 3D Tissue Morphogenesis In Vitro. Adv. Mater. Technol..

[22] Takahashi K., Tanabe K., Ohnuki M., Narita M., Ichisaka T., Tomoda K., Yamanaka S. (2007). Induction of Pluripotent Stem Cells from Adult Human Fibroblasts by Defined Factors. Cell.

[23] Masumoto H., Nakane T., Tinney J.P., Yuan F., Ye F., Kowalski W.J., Minakata K., Sakata R., Yamashita J.K., Keller B.B. (2016). The myocardial regenerative potential of three-dimensional engineered cardiac tissues composed of multiple human iPS cell-derived cardiovascular cell lineages. Sci. Rep..

[24] Miyazaki T., Isobe T., Nakatsuji N., Suemori H. (2017). Efficient Adhesion Culture of Human Pluripotent Stem Cells Using Laminin Fragments in an Uncoated Manner. Sci. Rep..

[25] Imai M., Iwatsuki-Horimoto K., Hatta M., Loeber S., Halfmann P.J., Nakajima N., Watanabe T., Ujie M., Takahashi K., Ito M. (2020). Syrian hamsters as a small animal model for SARS-CoV-2 infection and countermeasure development. Proc. Natl. Acad. Sci. USA.

