# Supplementary material for: Predicted risk of heart failure pandemic due to persistent SARS-CoV-2 infection using a three-dimensional cardiac model
Source: iScience. 2023 Dec 22;27(1):108641. doi: 10.1016/j.isci.2023.108641 (PMC10829886; doi:10.1016/j.isci.2023.108641)
Supplement: Document S1. Figures S1‒S3 [file mmc1.pdf]

iScience, Volume *27*

Supplemental information

Predicted risk of heart failure pandemic due to persistent SARS-CoV-2 infection using a three-dimensional cardiac model

Kozue Murata, Akiko Makino, Keizo Tomonaga, and Hidetoshi Masumoto

Supporting Information

Predicted risk of heart failure pandemic due to persistent SARS-CoV-2 infection using a three-dimensional cardiac model.

Kozue Murata[1,3], Akiko Makino[2], Keizo Tomonaga[2,*] & Hidetoshi Masumoto[1,3,*].

[1]Clinical Translational Research Program, RIKEN Center for Biosystems Dynamics Research, Kobe, Japan

[2]Laboratory of RNA Viruses, Department of Virus Research, Institute for Life and Medical Sciences, Kyoto University, Kyoto, Japan

[3]Department of Cardiovascular Surgery, Graduate School of Medicine, Kyoto University, Kyoto, Japan

*Corresponding authors

Lead contact

Hidetoshi Masumoto, MD, PhD. E-mail: hidetoshi.masumoto@riken.jp

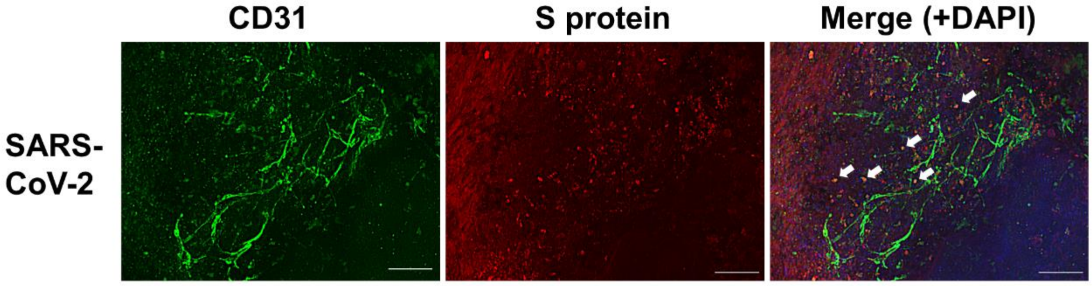

Supplemental Figure S1: Immunofluorescence analysis of CD31 and S protein for cardiac microtissues with persistent infection of SARS-CoV-2, related to Figure 1.

CD31: Green, S protein: Red. Nuclei were stained with DAPI (Blue). The analysis was performed after 17 days of infection. Arrows indicate co-expression of CD31 and S protein. Scale bars: 100 μm.

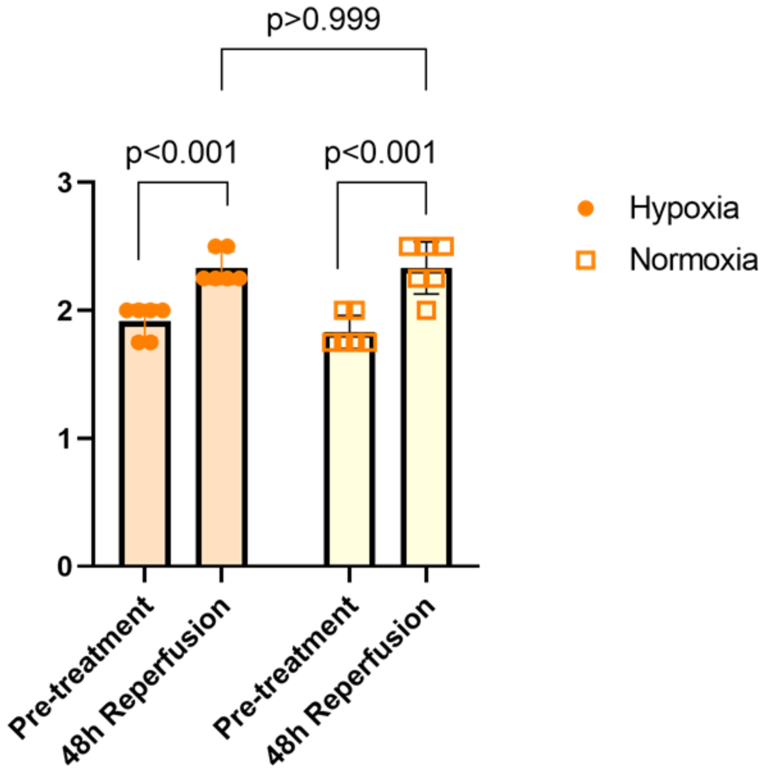

Supplemental Figure S2: SARS-CoV-2 titers in culture medium supernatant of cardiac microtissues with persistent infection of SARS-CoV-2 before and after hypoxia-reperfusion, related to Figure 2.

N=6 each, Error bars show S.D.

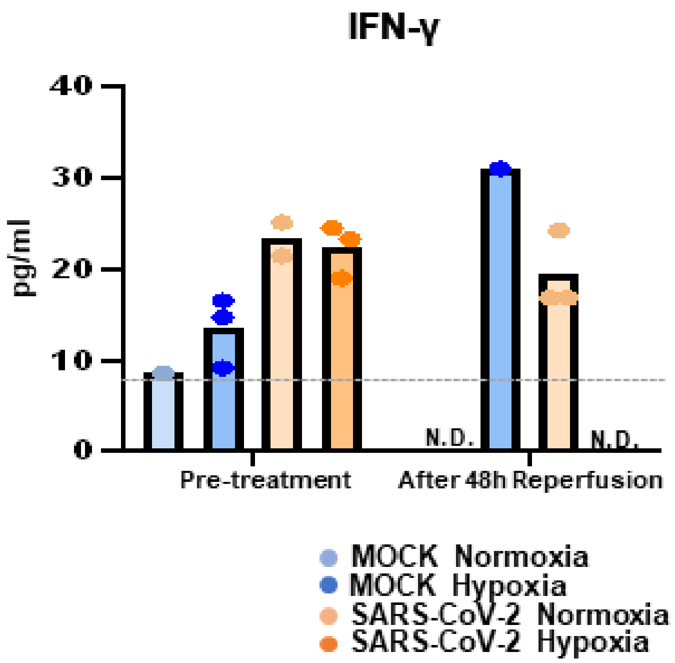

Supplemental Figure S3: Measurement of IFN-γ levels in the culture supernatant before and after hypoxia-reperfusion treatment in cardiac microtissues by ELISA, related to Figure 3.

Gray dashed lines represent the detection limits (5.69 pg/ml). The values under the detection limits are not shown. N.D. (Not Detected) values below the detection limit. Statistical analysis was not performed because the sample size exceeded the detection limit was limited (N=1-3).
